# Biophysical Insights into the Antitumoral Activity of Crotalicidin against Breast Cancer Model Membranes

**DOI:** 10.3390/ijms242216226

**Published:** 2023-11-12

**Authors:** Maria C. Klaiss-Luna, Juan M. Giraldo-Lorza, Małgorzata Jemioła-Rzemińska, Kazimierz Strzałka, Marcela Manrique-Moreno

**Affiliations:** 1 Chemistry Institute, Faculty of Exact and Natural Sciences, University of Antioquia, A.A 1226, Medellin 050010, Colombia; 2 Faculty of Biochemistry, Biophysics and Biotechnology, Jagiellonian University, Gronostajowa 7, 30-387 Krakow, Poland; 3 Malopolska Centre of Biotechnology, Jagiellonian University, Gronostajowa 7a, 30-387 Krakow, Poland

**Keywords:** crotalicidin, antitumoral peptide, lipid bilayer, breast cancer, membrane–peptide interactions, differential scanning calorimetry, Fourier-transform infrared spectroscopy

## Abstract

Bioactive peptides have emerged as promising therapeutic agents with antimicrobial, antifungal, antiparasitic, and, recently, antitumoral properties with a mechanism of action based on membrane destabilization and cell death, often involving a conformational change in the peptide. This biophysical study aims to provide preliminary insights into the membrane-level antitumoral mode of action of crotalicidin, a cationic host defense peptide from rattlesnake venom, toward breast cancer cell lines. The lipid composition of breast cancer cell lines was obtained after lipid extraction and quantification to prepare representative cell membrane models. Membrane–peptide interaction studies were performed using differential scanning calorimetry and Fourier-transform infrared spectroscopy. The outcome evidences the potential antitumoral activity and selectivity of crotalicidin toward breast cancer cell lines and suggests a mechanism initiated by the electrostatic interaction of the peptide with the lipid bilayer surface and posterior conformation change with membrane intercalation between the acyl chains in negatively charged lipid systems. This research provides valuable information that clears up the antitumoral mode of action of crotalicidin.

## 1. Introduction

Bioactive peptides (BAPs) are an extensive group of molecules that have emerged as potential therapeutic agents. These molecules exhibit a wide range of biological activities, including antimicrobial, antifungal, antiparasitic, antibiofilm, and, in the last decade, anticancer properties [1,2,3]. Their broad biological activity has been associated with an interaction with several molecular targets, particularly cell membrane, enzymes, and transmembrane receptors [4]. BAPs have been identified from countless sources, like plants, mammals, insects, and, one of the most interesting sources, venoms. Crotalicidin (Ctn) is a cathelicidin-related antimicrobial peptide isolated from the venom gland of the South American rattlesnake *Crotalus durissus terrificus*. Ctn is a cationic and amphipathic host defense peptide constituting a 34 amino acid sequence (KRFKKFFKKVKKSVKKRLKKIFKKPMVIGVTIPF), with a +15 net charge at a physiological pH from basic residues lysine and arginine [5,6]. The broad-spectrum activity of Ctn against ATCC strains and clinical isolates in several Gram-positive and Gram-negative bacterial species, such as *Staphylococcus aureus*, *Enterococcus faecalis*, *Pseudomonas aeruginosa*, *and Escherichia coli*, has been reported [6]. In recent publications, its antifungal and antichagasic activity has been evaluated in clinical strains of *Candida albicans* and in all morphological forms of the *Trypanosoma cruzi* parasite [7,8]. Regarding the antitumoral activity of Ctn, it is early research, but it has shown to be active in leukemia cell lines U937, THP-1, and MM6 and cervical cancer cells HeLa S3 with promising IC_50_ values lower than 3 μM [6]. However, the mechanism of action by which Ctn exerts its activity is still under study. Pérez-Peinado et al. suggested via a biophysical study that the bactericidal activity of the peptide is based on cell membrane disruption, which allows Ctn to cross into the lipid bilayer, inducing cell death [9].

It has been accepted that BAPs’ mechanism of action is based on the electrostatic interaction between the positively charged amino acids of the peptides and the negatively charged lipids of the bacterial membranes. For that reason, it has been suggested that BAPs often exhibit parallel antibacterial and antitumoral activity due to having similar targets such as negatively charged molecules in the cell membranes. Lipids such as phosphatidylglycerol, cardiolipin, lipoteichoic acids, and lipopolysaccharides are responsible for the interaction of peptides with bacteria, and therefore phosphatidylserine, sialylated gangliosides, O-glycosylated mucins, and heparin sulfates would explain this activity in tumoral cells [10,11,12]. 

Even when the antitumoral activity of Ctn has been reported, there are no biophysical reports regarding how the antitumoral activity of the peptide could be exerted, and therefore this issue is the main scope of this research. We are particularly interested in the potential antitumoral activity of Ctn toward breast cancer cells. Breast cancer is the most common cancer worldwide; it accounts for 1 in 4 new cancer cases and 1 in 6 cancer deaths [13]. Despite the available treatment options, there is significant controversy about the lack of selectivity and side effects from traditional chemotherapeutic agents, including the pharmacological resistance of cancer cells [14,15]. Scientific efforts to find new treatment approaches have focused on bioactive peptides. In fact, they are emerging as an innovative alternative due to their improved selectivity and the possibility of treating a malignant tumor already formed, a latent tumor, or a tumor at any growth rate without the need for being transported inside the cell because most of the peptides are membranolytic [15,16]. Biophysical studies would offer insights into the antitumoral mode of action at the membrane level with the aim to understand the molecular bases that rule the prospective membrane-peptide interaction. 

This study investigated the potential antitumoral mechanism of action and selectivity of Ctn toward breast cancer cells using biophysical techniques. In the first step, a phospholipid composition of MCF-7, MDA-MB-231 breast cancer cell lines, and spontaneously immortalized human keratinocytes (HaCaT) was determined using high-performance thin-layer chromatography (HPTLC). Afterward, lipid models were built to resemble the lipid composition of tumoral and non-tumoral membranes. Both systems were used to monitor the lipid–peptide interaction by means of differential scanning calorimetry (DSC) and infrared spectroscopy (FT-IR), where the thermodynamic properties, structure, and dynamics of the tumoral and non-tumoral model membranes were analyzed. Finally, the secondary structure–activity relationship of the peptide in specific aqueous and lipid environments was studied. The results of this work provide valuable information about the consequences of the lipid profile in the membrane–peptide interaction and contributes to clearing up the antitumoral mechanism of action of Ctn.

## 2. Results

### 2.1. Quantification of the Total Lipid Extracts of MDA-MB-231, MCF-7, and HaCaT Cell Membranes

**Figure 1:**
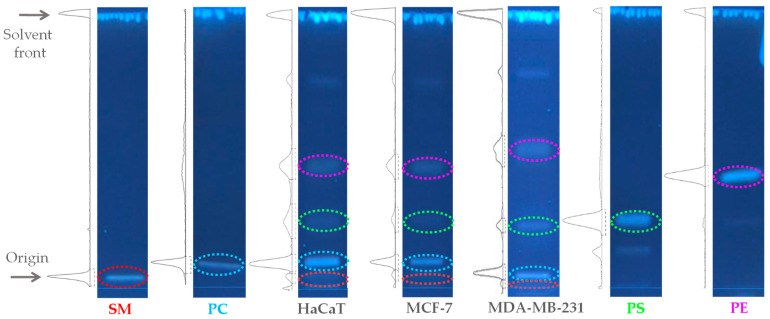
Densitometric chromatograms of lipid standards and total lipid extracts from HaCaT, MCF-7, and MDA-MB-231 cell lines. The solvent system was chloroform: methanol: petroleum ether: acetic acid (4:2:3:1 *v*/*v*). The plates were sprayed with primuline 0.05% in acetone:water (80:20 *v*/*v*). Abbreviations: PC, phosphatidylcholine; PS, phosphatidylserine; SM, sphingomyelin; and PE, phosphatidylethanolamine.

To achieve the preparation of representative multi-component lipid systems that mimicked the composition of the HaCaT, MCF-7, and MDA-MB-231 membranes, the total lipid extracts of each cell line were quantified using HPTLC [17]. The results of the quantification of the lipid profile for the three cell lines are summarized in Figure 1. Identification of lipid classes was established from their retardation factors in comparison with the corresponding standards. The HPTLC results indicate that the composition of HaCaT was PC/PE/SM/PS 47:27:5:21 (*w*/*w*); MCF-7, PC/PE/SM/PS 47:35:5:13 (*w*/*w*); and MDA-MB-231, PC/PE/SM/PS 43:33:4:20 (*w*/*w*). Based on the results obtained, we decided to omit PS from the synthetic non-tumoral model membrane HaCaT PC/PE/SM 47:27:5 (*w*/*w*) to mimic the zwitterionic character of non-tumoral cell membranes and to include it in the MCF-7 and MDA-MB-231 tumoral model membranes. The reason for this is that the PS content does not change significantly between epithelial and breast cancer cell lines. Instead, its distribution on the leaflets of the cell membrane is mainly altered in cancer [18].

### 2.2. Differential Scanning Calorimetry Experiments

#### 2.2.1. Evaluation of the Effect of Ctn on HaCaT, MCF-7 and MDA-MB-231 Model Membranes

To study the lipid–peptide interactions, DSC experiments were performed aimed at examining the effect of Ctn on the thermotropic behavior of the multi-component and individual lipid systems. Based on the calorimetric profiles obtained, the thermodynamic parameters associated with the change in state from the lamellar gel phase (L_β_) to the lamellar liquid crystalline phase (L_α_), such as the transition temperature (T_m_) and enthalpy (ΔH), were determined.

**Figure 2:**
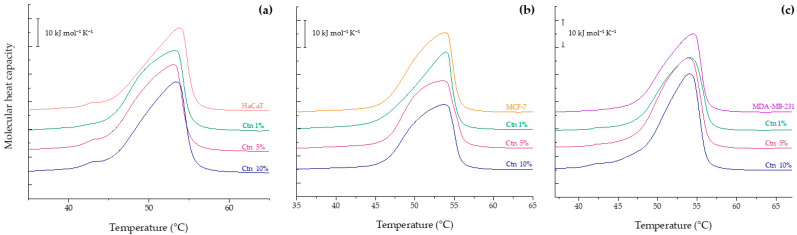
DSC heating thermograms of 1 mM model membranes (**a**) HaCaT, PC/PE/SM 47:27:5; (**b**) MCF-7, PC/PE/SM/PS 47:27:5:21; and (**c**) MDA-MB-231, PC/PE/SM/PS 43:33:4:20 with Ctn at 1, 5, and 10 mol%.

The thermogram of the non-tumoral model membrane mimicking HaCaT is shown in Figure 2a. The result shows a small peak at 42.93 °C, which represents a microdomain of DPPC molecules, accompanying a broad endothermic peak with a maximum at 53.79 °C and a ΔH = 36.94 kJ mol^−1^. The interaction with Ctn induced a mild shift in the T_m_ to lower temperatures at all the evaluated concentrations, as reported in Table 1. Even at the highest concentration of Ctn examined, the DPPC microdomain is still present in the thermogram, showing no higher perturbance of the lipid system. 

The calorimetric trace of the MCF-7 model membrane is presented in Figure 2b. It evidences a broad peak with a maximum at 53.86 °C and a phase transition enthalpy of 38.51 kJ mol^−1^, similar to that observed for HaCaT. The addition of the peptide had a remarkable effect on the peak height and a concomitant enthalpy reduction to ΔH = 33.48 kJ mol^−1^ at 10 mol%. Ctn also induced a mild decrease in the phase transition temperature at 5 and 10 mol%. The results of the thermal behavior of the MDA-MB-231 model membrane are represented in Figure 2c. The thermogram shows a broad endothermic peak located at 54.47 °C with a phase transition enthalpy of 41.47 kJ mol^−1^. The presence of the peptide at all the evaluated concentrations induced a new small shoulder located at 42 °C. The results highlight the selectivity of the peptide toward the tumoral model membranes MCF-7 and MDA-MB-231, where the lipid structure and physical properties were noticeably affected in comparison to the non-tumoral model membrane. This outcome suggests that Ctn disturbs the packing of the hydrophobic core in tumoral model membranes due to a possible intercalation between the hydrocarbon chains, decreasing the van der Waals interactions; meanwhile, in the non-tumoral model membrane, Ctn exerts its mild activity from the lipid surface.

#### 2.2.2. Evaluation of the Effect of Ctn on Individual Lipid Systems

**Figure 3:**
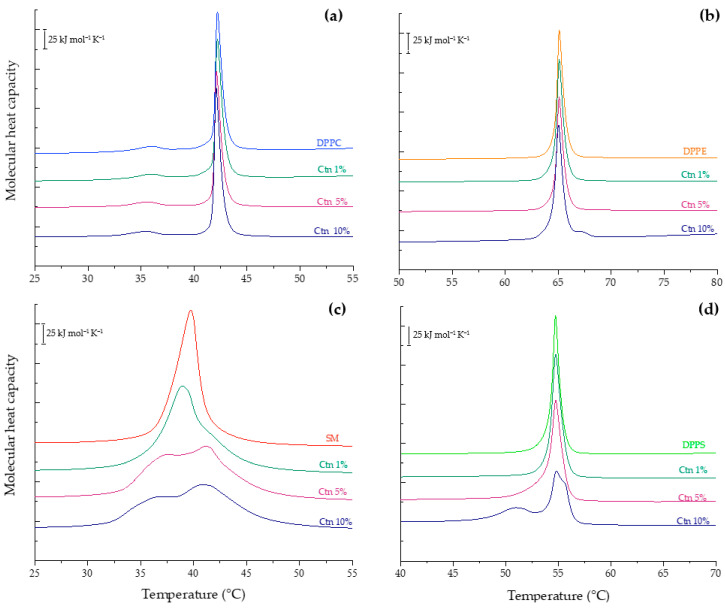
DSC heating thermograms of 1 mM multilamellar vesicles of pure (**a**) DPPC, (**b**) DPPE, (**c**) SM, and (**d**) DPPS containing Ctn at 1, 5, and 10 mol%.

Additional calorimetric experiments were performed using pure DPPC, DPPE, SM, and DPPS liposomes to obtain a comprehensive compilation of the effect of the peptide on the thermotropic behavior of the model membrane constituents. Phosphatidylcholine is the most abundant class of phospholipid in cell membranes, and the results of the incubation of Ctn with the DPPC liposomes in buffer are summarized in Figure 3a. The thermogram of the pure lipid system shows two distinct transitions. Centered at 36 °C with a transition enthalpy ΔH = 2.8 kJ mol^−1^ is a small endothermic but broad peak known as pretransition when the bilayer changes from the gel phase (L_β_) to the ripple phase (P_β′_), a periodically undulated bilayer related to changes in hydration of the bulky headgroup [19]. The second sharp and symmetric peak represents the main transition from the L_β_ to L_α_ phase with Tm = 42.2 °C and ΔH = 32.4 kJ mol^−1^. This result is in accordance with previous publications [20]. Then, the DPPC:Ctn mixtures were evaluated, and as shown in Figure 3a and Table 2, the peptide had no significant effect on the physicochemical parameters of the PC vesicles.

The lipid behavior of the hydrated DPPE vesicles in the presence of Ctn is presented in Figure 3b. The thermogram of the pure lipid liposomes shows a single highly cooperative transition at 65 °C with an enthalpy ΔH = 35.6 kJ mol^−1^. This result is in accordance with previous publications [21]. The addition of Ctn did not significantly change the calorimetric trace of the event and its related thermodynamic parameters, as reported in Table 2. The calorimetric profiles obtained for the SM:Ctn systems are summarized in Figure 3c. The thermogram of the pure SM shows a broad gel-to-liquid-crystalline transition centered at 39.7 °C with ΔH = 40.2 kJ mol^−1^. This result is in accordance with previous reports [22]. The incubation of the SM liposomes with the Ctn evokes profound changes in the DSC thermograms, indicating that the peptide exerts a destabilizing effect on the SM vesicles in a concentration-dependent manner by inducing phase separation, broadening the transition peak, and decreasing the enthalpy to 36.15 kJ mol^−1^ at the highest evaluated concentration. Finally, the DSC results of the PS liposomes in the presence of Ctn are presented in Figure 3d. The heating thermogram of the negatively charged PS liposomes showed a single and sharp transition peak at 54.7 °C with an enthalpy of 35.48 kJ mol^−1^. This result is in accordance with previous reports [20]. Upon the addition of Ctn, the fully hydrated PS liposomes show the reduction in height of the transition peak, and moreover, a phase separation at 10 mol% with additional peaks at 50.9 °C and 55.5 °C. This may suggest the formation of peptide-rich domains.

### 2.3. Phase Transition Measurements

Infrared spectroscopy was applied in combination with DSC to study the dynamics and structure of the lipids and peptides [23]. Infrared spectroscopy in ATR mode monitors the model membrane structure via the changes in the methylene symmetric stretching (υ_s_CH_2_) and carbonyl symmetric stretching (υ_s_C=O) vibrational modes, which describe the order of the fatty acid chains and hydration at the interface region, respectively. The analysis of the υ_s_CH_2_ vibration works as a sensor of the lipid order and packing of the model membranes. This parameter was analyzed via the shift in the maximum wavenumber position of the methylene symmetric stretching band from the hydrocarbon core upon temperature and peptide concentration. At low temperatures, where the lamellar gel phase prevails, the υ_s_CH_2_ lies around 2850 cm^−1^; meanwhile, at higher temperatures in the liquid-crystalline phase, the υ_s_CH_2_ is close to 2853 cm^−1^. Similarly, the ester carbonyl band is a sensitive marker of the system hydration and polarity at the interface structural region. The lipid bilayers evidence a hydration change in the υ_s_C=O wavenumber between 1740 cm^−1^ and 1735 cm^−1^ for the gel and liquid crystalline phases, respectively.

#### 2.3.1. Multi-Component Lipid Systems Using FT-IR

**Figure 4:**
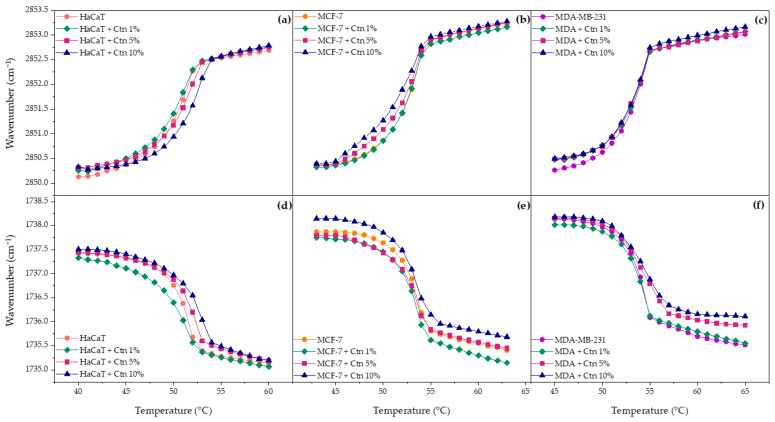
Methylene (**a**–**c**) and carbonyl (**d**–**f**) symmetric stretching peak position of HaCaT, MCF-7, MDA-MB-231 model membranes as a function of temperature with Ctn at 1, 5, and 10 mol%.

The results of the incubation of Ctn with the HaCaT lipid model are presented in Figure 4a. The pure lipid system is characterized by a sigmoidal curve whose inflection point corresponds to the transition temperature. The analysis of the results evidences how the wavenumbers increase as temperature increase due to the intra- and inter-molecular motion of hydrocarbon chains, disorder, and rapid trans-gauche rotational isomerism. Its interfacial hydration is also illustrated in Figure 4d and shows how the υ_s_C=O wavenumber decreases upon heating due to higher hydration from the lipid headgroups in the liquid crystalline phase. The interaction of Ctn with the HaCaT model membrane mildly affected the lipid phase transition by shifting the transition temperature to higher values, as a concentration-dependent effect. The above-mentioned finding is in concordance with the interfacial hydration results where the trend at all evaluated concentrations evidenced a slight shift in the υ_s_C=O wavenumbers toward higher values, indicating an overall release of water molecules from the lipid surface, due to lipid–peptide interaction. The more rigid the system, the fewer water molecules surround the phospholipid interface. 

The effect of Ctn on the lipid phase transition of the MCF-7 model membrane is presented in Figure 4b. The results showed that an increasing peptide concentration induced a mild drop in the T_m_, making the lipid system more fluid. Figure 4e shows that the peptide at 1 and 5 mol% slightly decreased the υ_s_C=O wavenumbers, suggesting higher hydration of the carbonyl groups. However, at 10 mol%, the opposite effect was observed. The overall results suggest that the way Ctn spans the hydrophobic core of the partially negative charged lipid bilayer occurs in a concentration-dependent manner. On the other hand, the effect of adding Ctn into the MDA-MB-231 lipid system did not induce remarkable changes in the lipid phase transition, as presented in Figure 4c. In contrast, the influence of the peptide on the interfacial region relied on dehydration, and it was more significant in the liquid crystalline phase.

#### 2.3.2. Individual Pure Lipid Systems Using FT-IR

**Figure 5:**
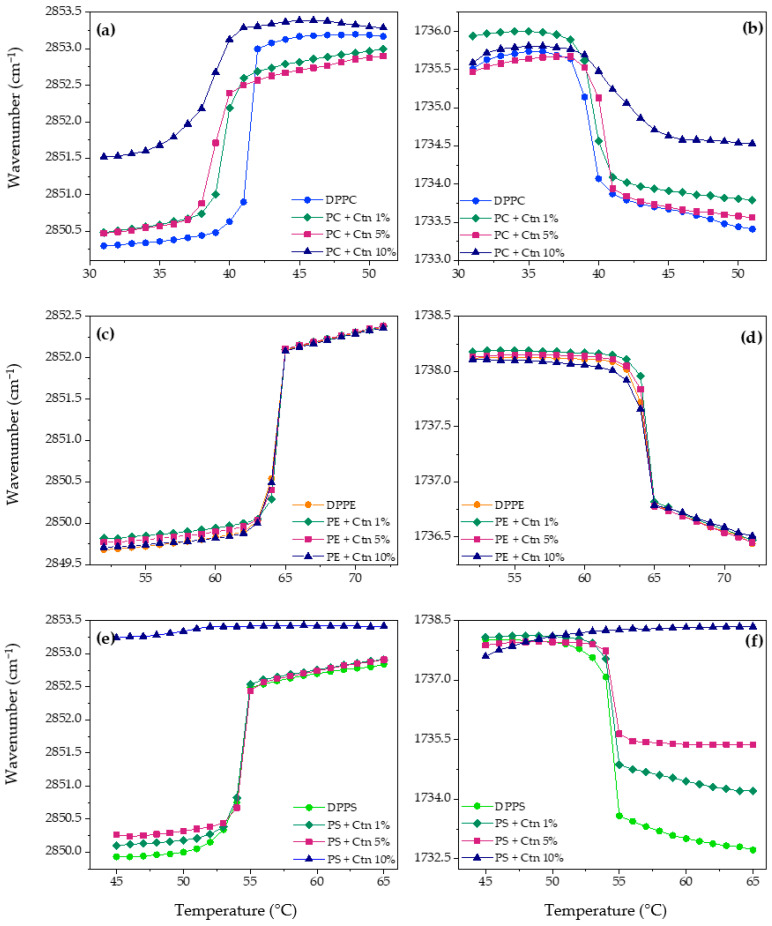
Methylene and carbonyl symmetric stretching peak positions of pure DPPC (**a**,**b**), DPPE (**c**,**d**), and DPPS (**e**,**f**) supported lipid bilayers as a function of temperature with Ctn at 1, 5, and 10 mol%.

Lipid packing and interfacial hydration were also analyzed in the supported lipid bilayers made by the pure lipids that were used as constituents of the eukaryotic model membranes. Figure 5 summarized the results of the exposition of the SLBs at different concentrations of Ctn. 

The results suggested that Ctn acts as a fluidizing agent on the SLBs made of DPPC (Figure 5a) with a more significant effect at 10 mol%. The previous results correlate with the dehydration effect detected in the analysis of the liquid crystalline phase in Figure 5b. In the case of DPPE, Figure 5c,d illustrate that the peptide has no significant effect on either the lipid order, packing, or interfacial hydration of pure phosphatidylethanolamine bilayers.

**Figure 6:**
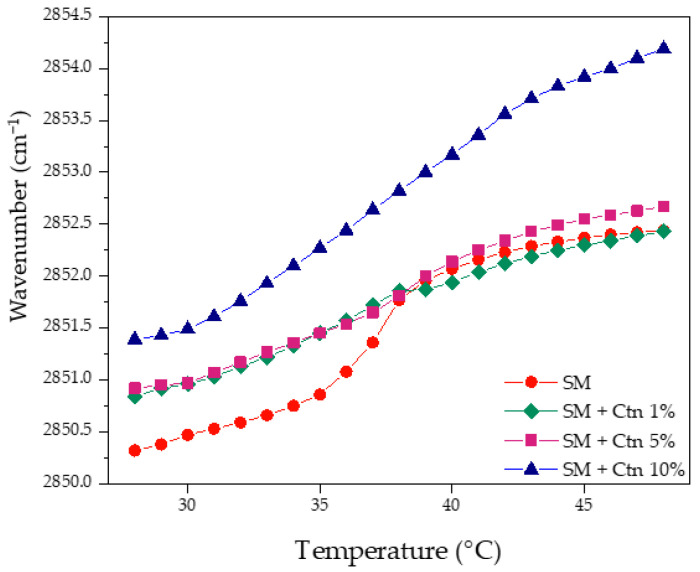
Methylene symmetric stretching peak position of pure SM supported lipid bilayers as a function of temperature with Ctn at 1, 5, and 10 mol%.

On the other hand, the strongest lipid–peptide interaction was evidenced with the negatively charged PS. Figure 5e,f show the mild fluidization of the υ_s_CH_2_ wavenumbers in the L_β_ phase and the progressive dehydration in the L_α_ phase until the complete abolishment of the phase transition at 10 mol%. The above suggests a strong electrostatic interaction between the cationic peptide and PS, leading to a complete perturbation of the lipid system. Similarly, data obtained for the SM-supported bilayers incubated with Ctn, illustrated in Figure 6, show that the peptide increases the system’s fluidity. This is occurring due to a suggested lipid packing disruption since there is a loss of cooperative melting according to the disappearance of the sigmoidal function shape. Interfacial hydration was not possible to follow because of the sphingomyelin chemical structure, where the only carbonyl group of the N-acyl chain is a weak hydrogen bond acceptor, making it difficult to follow the hydrogen bonding interactions.

### 2.4. Peptide Structure Prediction Methods

Ctn is a cathelicidin-related antimicrobial peptide from the South American rattlesnake *Crotalus durissus terrificus*. Ctn contains 34 amino acids that contribute with a +15 net charge at physiological conditions. Several reports have been conducted to establish a correlation between bioactive peptides’ secondary structure and their activity. It has been extensively suggested that as part of the interaction of a peptide with a membrane, they undergo a conformational change after membrane binding from a random to a helical secondary structure. This change depends on the peptide’s physicochemical properties like primary structure, net charge, hydrophobicity, amphipathicity, and concentration, but also membrane properties like lipid composition [24,25]. That is why in this study, Ctn’s conformational structure was analyzed using computational tools with static conditions and in solution experiments that were highly dynamic and specific in aqueous and lipid environments.

#### 2.4.1. De Novo Structure Prediction

**Figure 7:**
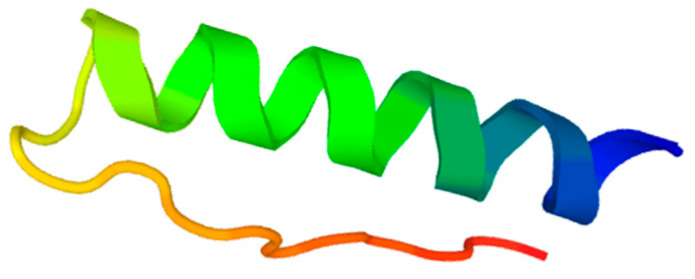
3D model of Ctn secondary structure predicted using PEP-FOLD3.

The online server PEP-FOLD3 predicted Ctn as an α-helix–coil peptide where the N-terminal sequence contributes with an α-helix. After the isoleucine located at position 21, a helix disruption ends with a disordered structure in the C-terminal segment (Figure 7) [26,27,28]. The computational results allow for the identification of a common conformational property of cathelicidins, often presenting a slight helical structure in aqueous solutions.

#### 2.4.2. BPROT1 Method

Secondary structure analysis using the BPROT1 method evidenced the slight α-helix component of Ctn in buffer between 10 to 14%. The results highlight the increase in the helical structure in the presence of specific lipid environments with respect to aqueous solution. Table 3 summarizes the conformational change in Ctn when interacting with pure lipids and model membranes. The more negatively charged the lipid system, the higher the conformational change. That is why the highest helical content of Ctn was observed in the MDA-MB-231 model membrane with 20% negative charge from PS, and in pure phosphatidylserine liposomes that were 100% negatively charged.

## 3. Discussion

Ctn is a 34-residue cationic host defense peptide isolated from the *Crotalus durissus terrificus* rattlesnake venom with demonstrated antimicrobial, antifungal, antichagasic, and antitumoral properties [6,7,8]. Previous biophysical studies proposed membrane disruption as the bactericidal mechanism of action; however, its antitumoral mode of action is still unexplored [9]. Bioactive peptides are at the zenith of drug development to become potential therapeutic agents for diseases, including cancer. This work was focused on understanding using biophysical experiments the potential antitumoral activity of Ctn in a breast cancer lipid model.

A critical starting point for this investigation was to achieve a representative multi-component lipid system that mimics the lipid composition of the implied cell membranes based on the most abundant phospholipids. The reported data describe as a general trend the phospholipid proportions PC > PE > PS > SM in mammalian cell membranes with feasible minor variations owing to the cell type [29]. The first step of the research was the densitometric scanning analysis of the lipid composition of the HaCaT, MCF-7, and MDA-MB-231 cell lines. The results follow the described general trend and highlight higher PE levels in tumoral than in non-tumoral lipid extracts. Shah et al. observed higher PE amounts in malignant breast cancer cells than in non-malignant cells, caused by the downregulation of ethanolamine kinase Etnk-1 [30]. In addition, Kim and collaborators identified higher levels of PE in MCF-7 than MDA-MB-231 and associated it with the estrogen receptor (ER) expression. The authors also evidenced an increased amount of PS in MDA-MB-231 compared to MCF-7, while PC and SM levels did not show significant differences, as in our results [31]. Unfortunately, there is a lack of data that compare the phospholipid composition of epithelial breast cancer cells with epidermal keratinocytes. Moreover, there is a general difference between tumoral and non-tumoral cells, namely the loss of lipid asymmetric distribution with the translocation of anionic PS from the cytoplasmic to exoplasmic leaflet in the membrane of malignant cells. As a result, the latter have a negative charge on the lipid surface in comparison to the mostly zwitterionic surface of non-tumoral cell membranes [32,33]. For this reason, we decided not to include PS in the HaCaT model membrane, to properly represent the differential factor.

In this study, the representative multi-component lipid systems were prepared to determine the effect of Ctn on the thermotropic and physicochemical properties of the membranes, such as lipid phase transition and packing. Moreover, we investigated the influence of the lipid composition of membranes on the conformation and activity of the peptide for insight into its mode of action. The DSC and FT-IR results evidenced that Ctn induced on the HaCaT model membrane a mild shift in T_m_ with a dehydration effect, while the enthalpy was not significantly affected. The above suggests a modest interaction of electrostatic forces and the proposed peptide location on the water–lipid interphase. Pašalić and collaborators demonstrated that cationic peptides will sense electrostatic interactions with zwitterionic lipid groups and possibly adsorb on the outer leaflet depending on the lipid membrane composition, ionic strength, and peptide concentration [34]. 

In the case of MCF-7, the complementary techniques illustrated a strong fluidization effect of the peptide owing to the reduction in the T_m_ and enthalpy values and the gain of water molecules at lower concentrations. Hence, we suggest that Ctn disturbs the packing of the lipid bilayer. It spans the hydrophobic core via an enthalpy-driven intercalation into the acyl chains, decreasing the van der Waals interactions, thus allowing the incorporation of more water molecules, and making the system more fluid. In general, cationic peptides interact rather with negatively charged lipid systems and exert a possible packing disruption of acyl chains, even more likely when they are longer than a 21 amino acid sequence [35,36]. The effects were similar but stronger in the case of Ctn incubated with the tumor MDA-MB-231 model membrane since additionally a phase separation was observed. This may suggest peptide aggregation and the formation of peptide-rich domains, thus providing evidence of a directly proportional relationship between the peptide intercalation and concentration [37]. The described effects of MDA-MB-231 were more appreciable via DSC than FT-IR, which can be related to the spontaneous curvature of the lipid vesicles in contrast to the flat supported lipid bilayers [38]. Curvature increases the permeability of the membrane for external molecules and contributes to detecting changes easily [38]. However, the binding event is closely followed using supported bilayers. We propose a charge neutralization between the cationic peptide and PS in the FT-IR outcome, which is why there was no major observation for MDA-MB-231. Nevertheless, we certainly highlight that complex lipid bilayer studies require complementary experimental approaches.

The membrane–peptide interaction results highlight the selectivity of Ctn toward the tumoral model membranes MCF-7 and MDA-MB-231, especially in the model with the highest PS content. The literature reports that bioactive peptides exhibit selectivity toward malignant cells and in a minor proportion against non-malignant cells, as supported by our results [39]. As a matter of fact, to understand the previous results and obtain a comprehensive compilation of Ctn’s effect, additional experiments were performed using pure lipid systems made of PC, PE, PS, and SM. The peptide exhibited a strong destabilizing effect on the SM vesicles in a concentration-dependent manner in the DSC and FT-IR studies. SM, through its sphingosine moiety, can establish several intra- and inter-molecular hydrogen bonds that change the lipid headgroups’ structure and interfacial region [40]. The results illustrated a strong destabilizing action by Ctn toward PS vesicles, inducing changes in the lipid microheterogeneity and promoting phase separation as a concentration-dependent effect. This suggests a lipid packing perturbation with possible intercalation and a reduction in van der Waals interactions. The above demonstrates the favorable electrostatic interaction between cationic Ctn and negatively charged PS, which was evidenced by the selective interaction with the mimicking models. The outcome is important since cancer cells report higher negative surface charge compared to normal cells.

As part of peptides’ mode of action, several authors have conducted research to set up a correlation between peptide structure and the extent of their biological activity, considering that bioactive peptides often undergo a conformational change after membrane binding. In this study, the online server PEP-FOLD3 and the infrared spectroscopy approaches evidenced the α-helix–coil conformation of Ctn in aqueous solutions and the increased helical content after membrane binding, especially in negatively charged lipid environments. Our results support the finding of Falcao and collaborators about the helical N-terminal and the disordered C-terminal sequence after exposure to helical-breaking proline, as well as the 26% helical content shift when Ctn was in the presence of anionic lipid vesicles made of phosphatidylglycerol mimicking the bacterial cell membrane [6]. The described α-helix–coil segment is reported to cause synergy in the antimicrobial activity of Ctn since the first structure element manages the phospholipid–peptide interaction and the second one the insertion depth [41,42]. Considering the above and our results, we suggest that there is a conformational change associated with the potential antitumoral activity of the peptide.

## 4. Materials and Methods

### 4.1. Reagents

The 1,2-Dipalmitoyl-*sn*-glycero-3-phosphocholine (DPPC, Lot 160PC-318), 1-,2-dipalmitoyl-*sn*-glycero-3-phosphoethanolamine (DPPE, Lot 160PE-106), 1-,2-dipalmitoyl-*sn*-glycero-3-phospho-L-serine sodium salt (DPPS, Lot 840037P-500MG-A-078), 1-palmitoyl-2-oleoyl-*sn*-glycerol-3-phosphocholine (POPC, Lot 850457P-500MG-A-211), 1-palmitoyl-2-oleoyl-*sn*-glycerol-3-phosphoethanolamine (POPE, Lot 850757P-500MG-B-151), 1-palmitoyl-2-oleoyl-*sn*-glycerol-3-phospho-L-serine sodium salt (POPS, Lot 840034P-25MG-397 A-250), and egg sphingomyelin (SM, Lot 860061P-25MG-A-116) were from Avanti Polar Lipids (Alabaster, AL, USA). The length of the acyl chains were selected for being the most abundant in the eukaryotic cells [43,44]. The N-2-hydroxyethylpiperazine-N’-2-ethanesulfonic acid (HEPES) was from Sigma-Aldrich (St. Louis, MO, USA). All other reagents of analytical grade were from Sigma-Aldrich.

The crotalicidin (Ctn, KRFKKFFKKVKKSVKKRLKKIFKKPMVIGVTIPF, Lot. U1440EE070-1/PE1314) was synthesized according to the sequence using the solid-phase method and purchased from GenScript (Piscataway Township, NJ, USA). Analytical HPLC determined the 95% purity, and the molecular weight was confirmed using MALDI–TOF mass spectrometry.

### 4.2. Cell Culture Conditions and Total Lipid Extraction

The breast cancer cell lines MCF-7 (Luminal A, ATCC HTB-22^TM^) and MDA-MB-231 (triple-negative, ATCC CRM-HTB-26^TM^) and human keratinocyte cell line HaCaT (non-tumoral, CLS 300493) were cultured in Dulbecco’s modified Eagle’s medium (DMEM), supplemented with 5% fetal calf serum, 100 µg/mL penicillin, and 100 µg/mL streptomycin. The cells were grown in a humidified incubator at 37 °C with 5% CO_2_/95% air. After checking under a microscope the proper growth, morphology, and adherence, the cells were trypsinized, pelleted, and washed with 2 mL of filtered and deionized water and centrifuged at 6000 rpm for 15 min at 4 °C in 15 mL falcon tubes. The supernatant was discarded, and the pellet was freeze-dried in a chamber at −60 °C, pressure < 260 μbar (SP Scientific, Gardiner, NY, USA).

Total lipid extraction was performed using the two-step Bligh and Dyer lipid extraction method, suitable for samples in medium, tissue, or cell suspensions [45]. The lyophilized cells were weighed in a clean test tube, resuspended in 5 mL of deionized water, and vortexed to guarantee homogenization. Then, the suspension was transferred into a clean glass separatory funnel and a 96 mL volume of the solvent mixture chloroform: methanol: water (1:2:0.8 *v*/*v*/*v*) was added. The system was shaken for 20 s after the solvent addition and then occasionally for about 18 h. Phase separation of the biomass–solvent mixture was performed by adding chloroform to obtain a final ratio chloroform: methanol: water (1:1:0.4 *v*/*v*/*v*). The chloroform phase was transferred into a 100 mL flask bottle for its concentration and washed 3 times with 0.9% NaCl (*w*/*v*). The organic phase was recovered and transferred into a 1.5 mL clean glass vial, where the solvent was evaporated under a nitrogen stream and vacuum. The extracts were kept at −20 °C before analysis [46].

### 4.3. High-Performance Thin-Layer Chromatography (HPTLC)

The total lipid extracts and lipid standards DPPC, SM, POPE, and POPS were dissolved in chloroform and sprayed semi-automatically using the sample dispenser CAMAG^®^ Linomat 5 (Muttenz, Switzerland) onto HPTLC plates (silica gel 60; Merck, Darmstadt, Germany) bandwise with a nitrogen stream. The plates were immersed into the developing chamber with eluent phase chloroform: methanol: petroleum ether: acetic acid (4:2:3:1 *v*/*v*/*v*/*v*) [17] and stained using the fluorescent dye primuline 0.05% in acetone: water (80:20 *v*/*v*). Then, the plates were photographed using a CAMAG^®^ TLC Visualizer 3 under lighting at 366 nm in the fluorescence mode. For quantification, densitograms and image profiles were generated and the data were processed using the winCATS 1.4.2 software (CAMAG^®^, Muttenz, Switzerland). Fluorescence scanning densitometry and the respective comparison with lipid standards (2.5 µg) quantified the phospholipid class content in the lipid extracts with a ±1% deviation.

### 4.4. Differential Scanning Calorimetry (DSC)

Stock solutions of DPPC and SM were prepared in chloroform and DPPE and DPPS in chloroform: methanol (70:30 *v*/*v*). For the DSC measurements, 1 mM MLVs of the individual lipids were prepared by adding the required volume from the stock into a glass test tube. In the case of non-tumoral and tumoral model membranes, 1 mM MLVs were prepared according to the lipid class content from the HPTLC results: HaCaT, DPPC/DPPE/SM 59:35:6 (*w*/*w*); MCF-7, DPPC/DPPE/SM/DPPS 47:35:5:13 (*w*/*w*); and MDA-MB-231, DPPC/DPPE/SM/DPPS 43:33:4:20 (*w*/*w*). The solvent was evaporated under a N_2_ stream, and the dried lipid film was hydrated using buffer (10 mM HEPES, 500 mM NaCl, and 1 mM EDTA pH 7.4). The MLVs were formed by vortexing the samples at a temperature above the main phase transition temperature of the lipid system for 6 min followed by 1 min sonication at the same temperature. The Ctn stock solution was prepared in the same buffer and added to the lipid film at 1, 5, and 10 molar%.

DSC measurements were performed using the Nano DSC Series III platinum capillary cell system (TA Instruments, New Castle, DE, USA). The sample cell was loaded with 400 μL of lipid or lipid:peptide suspensions and the reference cell was loaded with the same volume of buffer. The cells were sealed and equilibrated for 10 min at the starting temperature. Calorimetric analyses were carried out on samples with a pressure of 0.3 MPa. Heating/cooling rates were performed at 1 °C per minute and the scans were recorded in the temperature range of 15 to 60 °C for DPPC, 40 to 80 °C for DPPE, 15 to 50 °C for SM, 35 to 75 °C for DPPS, 15 to 65 °C for the HaCaT non-tumoral model membrane, and 15 to 75 °C for both tumoral model membranes, MCF-7 and MDA-MB-231. Heating scans were carried out first. For data processing, the reference scan was subtracted from the sample scan. Each data set was analyzed, and the values of transition temperature (T_m_) enthalpy (∆H) and entropy (ΔS) were calculated using the NanoAnalyze 3.12.0 software (TA Instruments, New Castle, DE, USA). At least three independently prepared samples were measured to check the reproducibility of the experiments. The experimental data were plotted using the Origin Pro 8.0 software (OriginLab Corporation, Northampton, MA, USA).

### 4.5. Fourier-Transform Infrared Spectroscopy (FT-IR)

20 mM lipid stock solutions of the individual lipids DPPC, SM, DPPE, and DPPS and multi-component lipid systems HaCaT, DPPC/DPPE/SM 59:35:6 (*w*/*w*); MCF-7, DPPC/DPPE/SM/DPPS 47:35:5:13 (*w*/*w*); and MDA-MB-231, DPPC/DPPE/SM/DPPS 43:33:4:20 (*w*/*w*) were prepared in chloroform and stored at −20 °C. Lipid transition measurements were performed in the BioATR II cell from the Tensor II spectrometer (Bruker Optics, Ettlingen, Germany) using an MCT (Mercury, Cadmium, Tellurium) detector, a spectral resolution of 4 cm^−1^, and 120 scans per measured temperature. The cell was connected to a circulating water bath Huber Ministat 125 (Huber, Offenburg, Germany) that controls the required temperature with ±0.1 °C accuracy. For the phase transition measurements of individual lipids, 20 µL of buffer 20 mM HEPES pH 7.4 was added directly to the silicon crystal of the cell to record the background in a programmed temperature ramp range from 30 to 52 °C for DPPC, 52 to 72 °C for DPPE, 28 to 48 °C for SM, and 41 to 61 °C/45 to 65 °C for DPPS with a heating rate of 1 °C/min and equilibration time of 120 s between each measurement. In the case of the tumoral and non-tumoral membranes, the buffer was 10 mM HEPES, 500 mM NaCl, and 1 mM EDTA, pH 7.4, and the temperature ramp range for the lipid systems was 40 to 60 °C for HaCaT, 43 to 63 °C for MCF-7, and 45 to 65 °C for MDA-MB-231. After recording the background, the supported lipid bilayers (SLBs) were prepared in situ on the crystal by adding 20 µL of the 20 mM lipid stock solution, the solvent was evaporated, and the lipid multilayer film was hydrated using 20 µL of the same buffer at a temperature above the T_m_ for 15 min for all lipid systems; then, the samples were analyzed under the same temperature range set as the background. During hydration, 1, 5, and 10 molar% of Ctn peptide were added in the same buffer.

Data processing was carried out using the OPUS 3D 7.5 software (Bruker Optics, Ettlingen, Germany), which automatically subtracted the background from each recorded sample. The lipid phase transition temperature was analyzed via the vibration of methylene groups (2970 to 2820 cm^−1^); for that, the frequency range of the symmetric extension (2850 to 2853 cm^−1^) was cut from the spectra with subsequent baseline correction using the 20% sensitivity rubber band method. The maximum wavenumber for the symmetric extension of each temperature was obtained using the peak picking tool. The same procedure was performed to analyze the hydration at the phospholipid interface region via the ester carbonyl stretching vibration around 1725 to 1740 cm^−1^. Finally, the data were plotted as a wavenumber as a function of temperature, and to determine T_m_, the experimental sigmoidal curve was fitted into a Boltzmann function with a Levenberg–Marquardt iteration algorithm to calculate the inflection point of the curve using the Origin Pro 8.0 software (OriginLab Corporation, Northampton, MA, USA) [38].

### 4.6. Peptide Structure Prediction Methods

#### 4.6.1. De Novo Structure Prediction of Ctn

The most suited cluster of Ctn at a neutral pH was assessed using the online server PEP-FOLD3. The peptide conformation was predicted based on its primary structure using the Hidden Markov Model Structural alphabet (SA-HMM) algorithm and a coarse-grained force field. After a local prediction and a model quality assessment, the best 3D model was generated for visualization [26,27,28]. 

#### 4.6.2. Conformational Study of Ctn

Six mM SUVs of POPC, POPS, and models HaCaT, POPC/POPE/SM 59:35:6 (*w*/*w*); MCF-7, POPC/POPE/SM/POPS 47:35:5:13 (*w*/*w*); and MDA-MB-231, POPC/POPE/SM/POPS 43:33:4:20 (*w*/*w*) were prepared. Each lipid mass was weighed, added into a 1.5 mL clean glass vial, mixed, and dissolved in chloroform. The solvent was evaporated under a nitrogen stream; the lipid films were hydrated with buffer 10 mM HEPES, 500 mM NaCl, and 1 mM EDTA, pH 7.4, and put into a sonicator bath (Elma Schmidbauer GmbH, Singen, Germany) at 50/60 Hz and at a temperature above the main phase transition temperature of the lipid system for 30 min. The lipid suspensions and peptide solutions prepared at 3 mg/mL in the same buffer were at 37 °C in a digital dry bath (Thermo Fisher Scientific™, Waltham, MA, USA). To determine the secondary structure, the liposomes and peptide solutions were mixed to attain a peptide 15 molar% concentration. The experiments were performed at 37 °C by an AquaSpec transmission cell (Bruker Optics, Ettlingen, Germany) integrated with the Tensor II spectrometer and using 124 scans per spectrum. The secondary structure element content of α-helix was predicted using the BPROT1 method supplied by the Confocheck^TM^ system (Bruker Optics, Ettlingen, Germany), which calculates the secondary structure content with a multivariate partial least squares algorithm (PLS) based on a calibration data set of 45 proteins with a ±4% deviation.

## 5. Conclusions

This study offers preliminary data about the potential antitumoral activity of Ctn in breast cancer from a biophysical point of view. Several factors influence the activity of the peptide including its primary structure, the +15 net charge, amphipaticity, and concentration. However, it was also demonstrated that the differences in the lipid profile of tumoral and non-tumoral cells have considerable consequences on the membrane–peptide interaction that modulates the binding and activity of Ctn. This work suggests an initial electrostatic interaction of the peptide with the lipid bilayer surface and posterior membrane disruption of negatively charged systems due to possible intercalation between the acyl chains. The above agrees with the bactericidal mode of action proposed by Pérez-Peinado and co-workers and confirms that Ctn is a membrane-active peptide [9].

## Figures and Tables

**Table 1:** Thermodynamic parameters of main phase transition and pretransition of representative tumoral and non-tumoral multilamellar liposomes without and with Ctn at 1, 5, and 10 mol%. Data were determined from heating scans collected at a rate of 1 °C min^−1^. The main phase transition temperature and enthalpy accuracy were ±0.01 °C and ±0.8 kJ/mol, respectively.

	Heating		
	T [°C]	ΔH [kJ mol^−1^]	ΔS [kJ mol^−1^ K^−1^]
HaCaT *	42.93 and 53.79	36.94	0.11
+1 mol% Ctn	53.21	39.36	0.12
+5 mol% Ctn *	43.21 and 53.05	41.50	0.13
+10 mol% Ctn *	43.16 and 53.39	41.99	0.13
**MCF-7**	53.86	38.51	0.12
+1 mol% Ctn	53.94	34.14	0.10
+5 mol% Ctn	53.51	35.32	0.11
+10 mol% Ctn	53.67	33.48	0.10
**MDA-MB-231**	54.47	41.47	0.13
+1 mol% Ctn *	42.07 and 54.01	42.77	0.13
+5 mol% Ctn *	42.20 and 53.91	40.24	0.12
+10 mol% Ctn *	42.50 and 54.03	39.64	0.12

* 2 or more phases.

**Table 2:** Thermodynamic parameters of main phase transition and pretransition (#) of pure, fully hydrated DPPC, DPPE, SM, and DPPS multilamellar liposomes and their lipid/peptide mixtures determined from heating scans collected at a heating rate of 1 °C min^−1^. The main phase transition temperature and enthalpy accuracy were ±0.01 °C and ±0.8 kJ/mol, respectively.

	Heating		
	T [°C]	ΔH [kJ mol^−1^]	ΔS [kJ mol^−1^ K^−1^]
**DPPC**	42.20 35.84 (#)	32.37 2.84	0.10 0.01
+1 mol% Ctn	42.11 35.26	28.86 1.65	0.09 0.01
+5 mol% Ctn	42.12 35.57	29.05 2.38	0.09 0.01
+10 mol% Ctn	42.08 35.40	30.92 2.39	0.10 0.01
**DPPE**	65.04	35.60	0.11
+1 mol% Ctn	65.07	33.61	0.10
+5 mol% Ctn	65.07	30.46	0.09
+10 mol% Ctn	65.02	36.32	0.11
**SM**	39.73	42.00	0.13
+1 mol% Ctn	38.93	38.93	0.12
+5 mol% Ctn *	37.5 and 41.13	38.27	0.12
+10 mol% Ctn *	36.82 and 40.84	36.15	0.12
**DPPS**	54.69	35.48	0.11
+1 mol% Ctn	54.73	36.62	0.11
+5 mol% Ctn	54.72	38.28	0.12
+10 mol% Ctn *	50.93, 54.78 and 55.49	34.29	0.10

* Two or more phases.

**Table 3:** Prediction of the conformational change in Ctn in liposomes of pure POPC, POPS, and model membranes HaCaT, MCF-7, and MDA-MB-231 at 37 °C. The helical increase percentage was calculated with respect to buffer and has a deviation of ±4.4%.

System	Helical Increase (%) *
**POPC**	3.8
**HaCaT**	12.5
**MCF-7**	10.2
**MDA-MB-231**	13.7
**POPS**	46.0

* with respect to buffer.

## Data Availability

Data are contained within the article.
